# Left-Handedness in Professional and Amateur Tennis

**DOI:** 10.1371/journal.pone.0049325

**Published:** 2012-11-07

**Authors:** Florian Loffing, Norbert Hagemann, Bernd Strauss

**Affiliations:** 1 Institute of Sports and Sports Science, University of Kassel, Kassel, Germany; 2 Institute of Sport and Exercise Sciences, University of Münster, Münster, Germany; Universidad Europea de Madrid, Spain

## Abstract

Negative frequency-dependent effects rather than innate predispositions may provide left-handers with an advantage in one-on-one fighting situations. Support mainly comes from cross-sectional studies which found significantly enhanced left-hander frequencies among elite athletes exclusively in interactive sports such as baseball, cricket, fencing and tennis. Since professional athletes’ training regimes continuously improve, however, an important unsolved question is whether the left-handers’ advantage in individual sports like tennis persists over time. To this end, we longitudinally tracked left-hander frequencies in year-end world rankings (men: 1973–2011, ladies: 1975–2011) and at Grand Slam tournaments (1968–2011) in male and female tennis professionals. Here we show that the positive impact of left-handed performance on high achievement in elite tennis was moderate and decreased in male professionals over time and was almost absent in female professionals. For both sexes, left-hander frequencies among year-end top 10 players linearly decreased over the period considered. Moreover, left-handedness was, however, no longer seems associated with higher probability of attaining high year-end world ranking position in male professionals. In contrast, cross-sectional data on left-hander frequencies in male and female amateur players suggest that a left-handers’ advantage may still occur on lower performance levels. Collectively, our data is in accordance with the frequency-dependent hypothesis since reduced experience with left-handers in tennis is likely to be compensated by players’ professionalism.

## Introduction

The polymorphism in human handedness just as the relative rarity of left-handers compared to right-handers persists since thousands of years [1], [2]. While recent reports suggest that 10–13% of the western population is left-handed [3], percentages vary, among others, depending on the item used to assess someone’s handedness. For example, according to a large survey the preference of people aged 18–40 years for throwing left-handed ranged from 9.50% to 10.85% in men and from 6.93% to 7.99% in women, respectively, whereas left-handedness for writing was markedly higher (men: 12.14%–14.13%; women: 9.72%–11.85%) [4]. An almost consistent finding across studies is the stronger tendency for left-handedness in males compared to females [5]. A by now unsolved question is how the handedness polymorphism could be maintained despite left-handedness potentially being linked with negative traits such as a higher risk to suffer from health disorders [6]. Negative frequency-dependent selection mechanisms might explain this phenomenon. More specifically, analogous to survival strategies observed in the animal kingdom [7], [8], left-handers might have benefited from a fitness advantage in one-on-one fighting situations due to being rarer compared to right-handers, which, in turn, helped compensate for some of the costs inherent to left-handedness [9].

In support of this view, a significant excess of left-handed athletes, particularly in male competition [3], [10], was found repeatedly in the high echelons of sports that are characterized by direct interactions between contestants (e.g. 20% to 40.6% in baseball) [3], [11], [12]; however, not in sports that are non-interactive (e.g. darts) [13]. This consistent pattern indicates that the characteristics underlying interactive sports favour left-handed performers rather than specific mechanisms inherent to left-handedness such as right hemisphere specialization [3], [10], [14]. Recent laboratory- and field-based research provided evidence as to the perceptual-cognitive mechanisms underlying a left-handers’ negative frequency-dependent advantage: outcomes of left-oriented actions were harder to predict than more experienced right-oriented actions (e.g. stroke direction in tennis) [15], [16] and players did not mirror their game-play behaviour when faced with left-handed opponents [17].

A shortcoming of previous handedness distribution research is that predominantly cross-sectional designs were used or that data from several years was combined [14]. Tracking handedness distribution continuously over time, however, is vital for better understand the impact of handedness on expert performance [18]. To exemplify, in Major League Baseball frequencies of left-handed pitchers and batters almost logarithmically increased over decades from 1876 to 1985 and stabilized at a distinct overrepresentation around 30% [19]. Interpretation of this pattern as an evolutionary stable strategy makes sense in team sports since being able to resort to a considerable repertoire of left- and right-handed players allows a flexible team strategy [20]. Similarly, small teams such as tennis doubles are assumed to have a performance advantage if they are composed of one right- and one left-handed player [21]. In individual sports like tennis singles, however, team-strategic aspects do not come into play at all since performance solely depends on the two individuals involved in competition. Time-dependent fluctuations in left-hander frequencies in tennis therefore should stem from basic processes related to learning and potential adaptation to left-handed opponents.

To this end, we longitudinally tracked left-hander frequencies in professional tennis singles (Study 1) and contrasted these data with left-hander frequencies in a recent sample of non-professionals (Study 2). Since in the course of their career elite athletes spend thousands of hours with training and competition to develop and maintain their excellence [22], one might assume that the predominant confrontation with right-handed opponents reinforces the left-handers’ negative frequency-dependent advantage, which results in an almost robust excess of left-handers among elite tennis players over time [23]. However, athletes’ continuous performance improvements due to increased deliberate practice activities during expertise development [24], higher intensity and quality of training regimes [25] as well as the availability of statistics on competitors’ past performances may help professionals circumvent the detrimental negative frequency-dependent effects nowadays. We expected that this would be reflected in an interim excess of left-handers among elite performers only. Moreover, we hypothesized that in contrast to the early years in professional tennis left-hander frequencies would no longer increase with better ranking position [10]. Previous work on laterality effects in male and female sporting professionals found handedness to be performance-relevant in male rather than in female competition [3], [10], [12], [26]. Therefore, we expected the above hypothesized development to be more pronounced in men’s as opposed to ladies’ tennis.

## Study 1: Method

We analysed handedness distribution in female and male professional players listed in year-end world rankings from 1975 to 2011 (ladies) and 1973 to 2011 (men) as well as in players participating at the four Grand Slam tournaments (i.e. Australian Open, French Open, Wimbledon, and US Open) from the beginning of the open era in 1968 to 2011 (Table 1 & 2). Data on rankings, tournaments and players’ handedness were obtained from various open-source online databases (e.g. http://www.atpworldtour.com, http://www.wtatennis.com, http://www.itftennis.com, http://www.scoreshelf.com/en/tennis/) and from searches on the web (e.g. pictures or videos of players). Ethics approval was not considered necessary and therefore not obtained for this study since we examined data on public figures which is freely available online [27].

**Table 1:** Summary statistics on handedness in men’s professional tennis.

				Handedness (%)		
Dataset	Category	N	N/A	Left	Right	Ambidextrous
Year-end rankings (1973–2011)	All players	3746	–	9.58	90.23	0.19
	Top 100	843	–	13.40	86.36	0.24
	Top 10	116	–	13.79	86.21	–
	World no. 1	16	–	18.75	81.25	–
Grand Slams (1968–2011)	All players (first rounds)	1926	5	10.88	88.96	0.16
	Finalists	99	–	17.17	82.83	–
	Runner-up	85	–	17.65	82.35	–
	Winner	52	–	21.15	78.85	–

This table shows the handedness distribution for male professional tennis players in different datasets and categories. The column “N/A” indicates the number of players whose handedness for playing tennis was not available.

**Table 2:** Summary statistics on handedness in ladies’ professional tennis.

				Handedness (%)		
Dataset	Category	N	N/A	Left	Right	Ambidextrous
Year-end rankings (1975–2011)	Top 100	827	93	8.58	91.42	–
	Top 10	86	–	8.14	91.86	–
	World no. 1	10	–	20	80	–
Grand Slams (1968–2011)	All players (first rounds)	1631	602	9.23	90.48	0.29
	Finalists	69	–	8.70	91.30	–
	Runner-up	58	–	8.62	91.38	–
	Winner	41	–	9.76	90.24	–

This table shows the handedness distribution for female professional tennis players in different datasets and categories. The column “N/A” indicates the number of players whose handedness for playing tennis was not available.

### Results and Discussion

#### Year-end rankings

**Figure 1:**
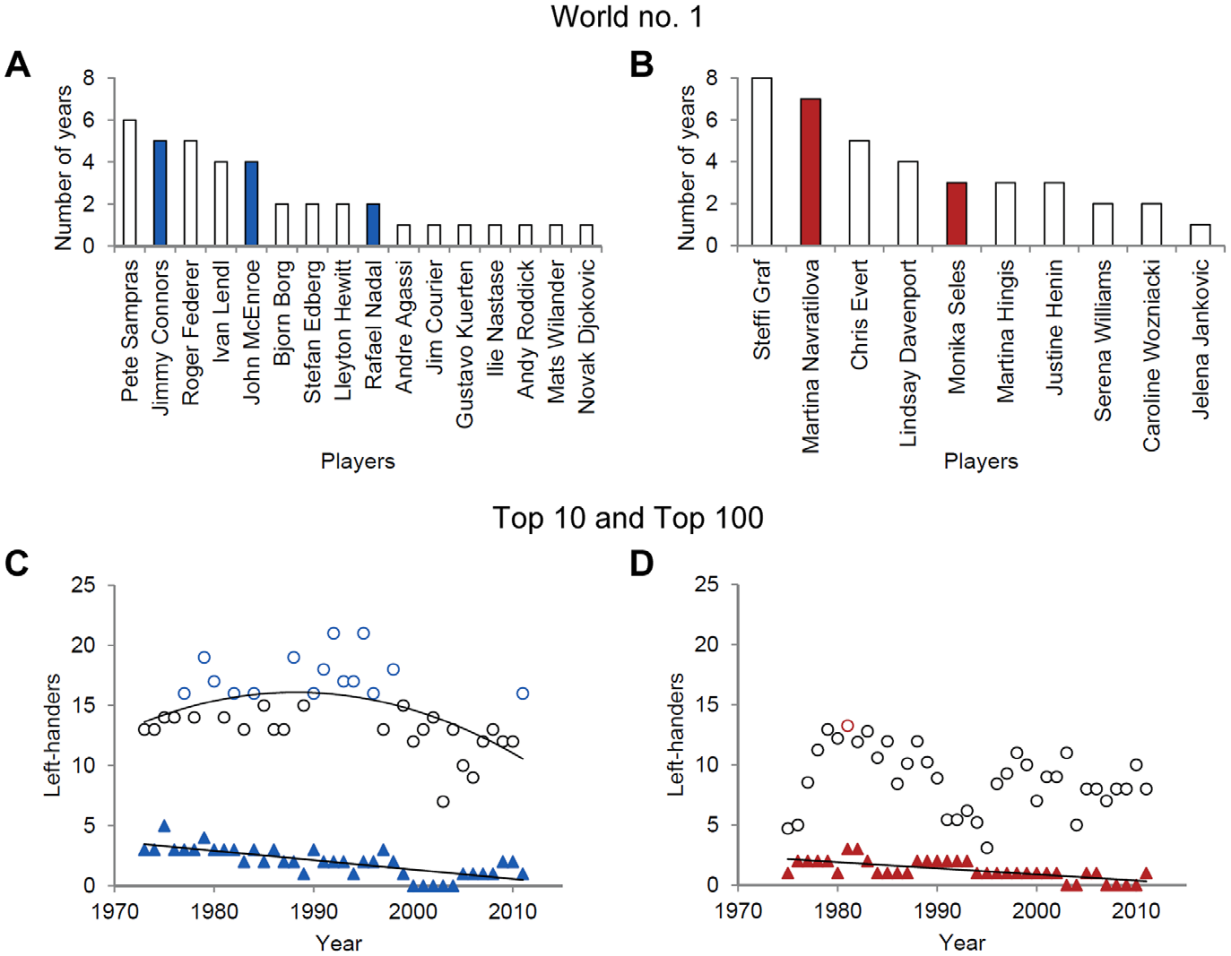
Left-handedness in year-end rankings. Number of years that individual players were ranked as world no. 1 in (A) men’s (1973–2011) and (B) ladies’ (1975–2011) year-end rankings. Coloured bars denote left-handed players. Note that two female players were listed as world no. 1 in 1995 (Monica Seles and Steffi Graf). Frequencies of (C) male and (D) female left-handed performers in the top 100 (circles) and top 10 (triangles) rankings over time. For men’s tennis, data was fitted to second-order (top 100, *R* ^2^ = .26) and first-order polynomials (top 10, *R* ^2^ = .57), respectively. Blue and red coloured circles indicate a significant (*p*<.05) excess of left-handed players in men’s and ladies’ top 100 rankings, respectively, compared to the normal population. Note that for female professionals, relative frequencies of left-handed players in top 100 rankings are illustrated because of some missing handedness data (see Table S1). Data of top 10 female players was fitted to a first-order polynomial with *R* ^2^ = .48.

In men’s professional tennis three out of 16 different year-end world no. 1 players were left-handed and these players hold that position in 11 of 39 years (Fig. 1A). Differentiation of left-hander frequencies by year revealed a linear decline in top 10 performers, whereas in top 100 players frequencies first increased and then decreased as reflected in a second-order polynomial (Fig. 1C). With regard to female professionals, 10 different players were listed as year-end world no. 1 and two of them played tennis left-handed (Fig. 1B). The two left-handers occupied that position in 10 of the 37 years considered. Here also left-hander frequencies in top 10 players linearly declined over the years. However, there was no clear pattern in the evolution of left-hander frequencies among the best 100 players in year-end rankings (Fig. 1D and Table S1).

Analogous to previous research [3], [12] and following the procedure described by [3], for each year-end ranking we calculated one-tailed 2×2 Fisher exact tests to check the prediction that left-handedness in male and female top 100 performers would be significantly increased compared to the normal population. Reference values for observed handedness distribution in the normal population were obtained from a large survey that examined, among others, handedness for writing and throwing across different age groups [4]. We chose handedness data on people aged 18–30 years (see also [3]) because that age group was considered near to the age range in top 100 tennis professionals. Since handedness for holding a racket and throwing are closely connected [28] we compared observed handedness in tennis professionals (see Table S1 for a detailed list) with observed handedness for throwing (men: *n*_LH_ = 8035, *n*_RH_ = 72489; ladies: *n*_LH_ = 8748, *n*_RH_ = 107915) [3], [29]. The tests were arranged such that the *p*-values indicated the probability for finding more or an equal number of left-handed professionals by chance given the normal population data. As to men’s tennis, analyses revealed a significant (*p*<.05) excess of left-handers in 15 out of the 39 years considered and, as was indicated by the inverse U-shaped pattern in left-hander frequencies across time, in the 1990s in particular (see Fig. 1C). Female left-handed professionals were significantly overrepresented only in the year-end ranking of 1981 (*p* = .046; Fig. 1D; see Table S1 for a list of all *p*-values and additional information).

#### Ranking interval and left-hander frequencies in men’s tennis

In addition to the investigation of left-hander frequencies among the top 10 and top 100 performers, we checked the data of the top 500 players in year-end rankings, where available, for an association between playing left-handed and high sporting achievement (see Table S2 for additional information). These analyses, however, were restricted to men’s tennis because handedness was almost impossible to assess for female professionals ranked below the top 100 and for the early years in particular.

**Figure 2:**
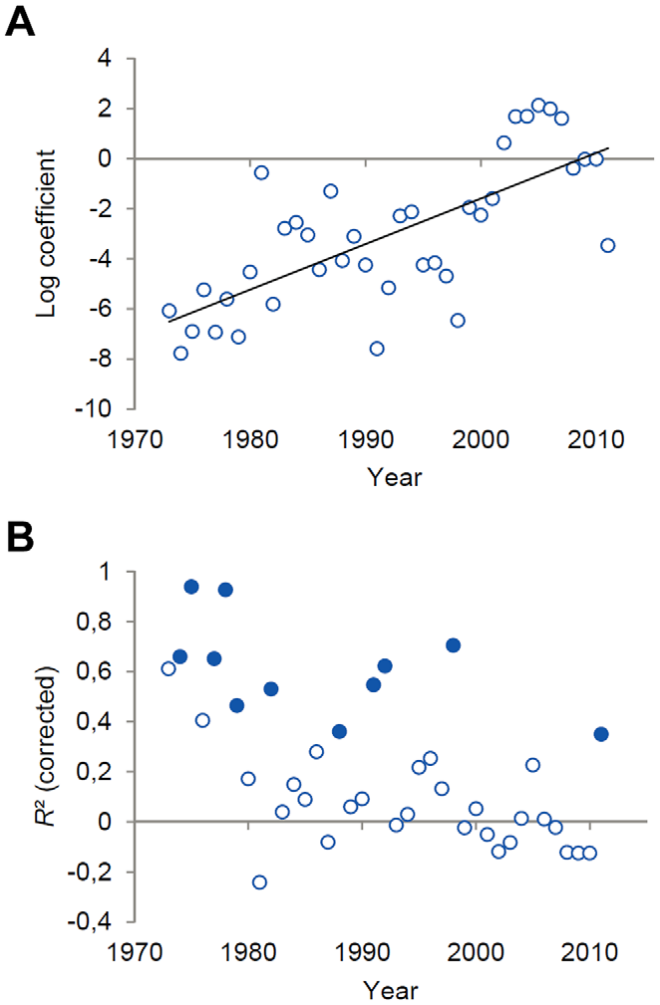
Results from logarithmic fittings of left-hander frequencies to ranking intervals. (A) Coefficients obtained from the logarithmic fittings of left-hander frequencies to ranking intervals of top 500 players in year-end rankings. Note that for the years 1973 to 1976 and for 1981 only data of the top 250 to top 400 male players was available (see Table S2). Ranking intervals were considered if the number of players whose playing hand was known was larger than or equal to 20. Higher (lower) proportion of left-handed players with better ranking interval is reflected in negative (positive) coefficients. Coefficient data was fitted to a first-order polynomial with *R* ^2^ = .51. (B) Explained variance (corrected *R* ^2^) in left-hander frequencies by ranking interval across the period considered. Blue filled circles mark the years where the explained variance was significantly different from 0 (*p*<.05).

We fitted the percentage of left-handed players found in ranking intervals of 50 players (i.e. 1 =  top 50 players, 2 = 51−100, …, 10 = 451−500) to logarithmic functions for each year-end ranking. We used logarithmic fittings based on previous findings on handedness distribution in tennis rankings [10]. Moreover, if playing left-handed provided a performance advantage, we would expect that left-hander frequencies are highest in ranking intervals representing the best players in year-end rankings (i.e. top 50) and that these frequencies asymptotically decrease (i.e. stabilize) at ranking intervals representing “low” achievers among professionals (e.g. 400–450 and 451–500). Negative coefficients obtained from the fittings represent an increase in left-hander frequencies with better ranking interval, thus indicating a performance advantage for left-handers. Conversely, positive coefficients would result from a decrease in left-hander frequencies with better ranking interval, which would indicate, similar to coefficients close to zero, that performing left-handed does not provide an advantage in terms of high achievement in year-end world rankings. Here, the time-dependent course of coefficients, as illustrated in Fig. 2A, supports our prediction that the left-handers’ advantage would decrease over time. Performing left-handed was, but no longer is, associated with higher probability of attaining high echelons in men’s professional world rankings. Note, however, that the explained variance revealed through logarithmic fittings varied over the years and that the corresponding values for *R* ^2^ were significantly (*p*<.05) different from zero in about 25% of the cases (Fig. 2B).

#### Grand Slam tournaments

**Figure 3:**
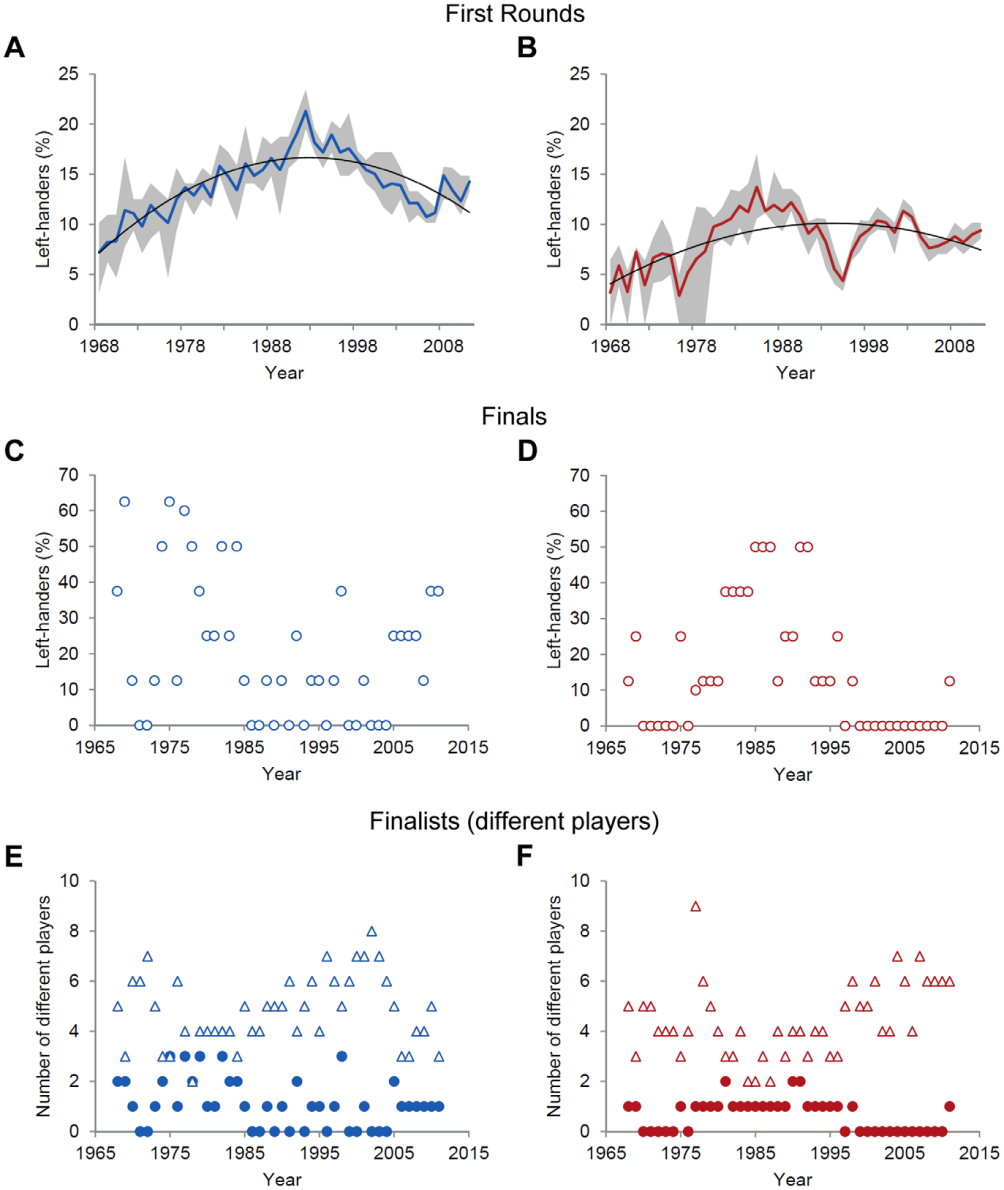
Left-handedness at Grand Slam tournaments (1968–2011). Mean percentages of left-handed players in the first rounds of (A) men’s and (B) ladies’ singles competition (both fitted to second-order polynomials, men: *R* ^2^ = .76, ladies: *R* ^2^ = .39). Grey shaded areas indicate the range (min to max) of left-hander frequencies across the tournaments per year. Note that for 1977 five instead of four tournaments were included since the Australian Open was hold twice in January and December. Percentages of (C) male and (D) female left-handed players in Grand Slam finals per year. Number of different Grand Slam finalists by playing handedness for (E) men and (F) ladies. Filled circles represent left-handed players, triangles represent right-handed players.

With regard to the most prestigious tennis tournaments in the world, we determined the frequency of left-handed players participating in the first rounds and in the finals of men’s and ladies’ competition for each tournament and for each year (Tables S3 & S4). To obtain representative values for left-hander frequencies among first round players we averaged those values across the tournaments for each year. The resulting left-hander frequencies showed similar patterns to those found in the top 100 of year-end rankings for both male (Fig. 3A) and female (Fig. 3B) professionals (correlation between left-hander frequencies in top 100 and in first round players, men: *r* = .62; ladies: *r* = .57). This similarity is plausible since the best ranked players in the world predominantly participate at these events.

In men’s tennis the frequencies of left-handed players among the finalists of Grand Slam tournaments were highest in the early years and decreased over time (Fig. 3C). In ladies’ tennis the highest incidence of left-handed finalists was reached in the mid of the analysed period (Fig. 3D), which, however, was due to the high performances of two left-handed female players only (Martina Navratilova: 1985–1987 and 1991; Monica Seles: 1991-1992). Overall and in recent years in particular the number of different left-handed finalists was considerably lower than the number of different right-handed players in men’s (Fig. 3E) and ladies’ competition (Fig. 3F). To exemplify, Rafael Nadal was the only left-handed male player who participated in Grand Slam finals from 2006 to 2011 (Fig. 3E; Table S5) and no left-handed female professional made it into any of these finals from 1999 to 2010 (Fig. 3F; Table S6).

Collectively, as judged from left-hander frequencies and compared to other interactive sports [3], [14], the left-handers’ advantage in professional tennis appears moderate in men’s and low, if at all, in ladies’ competition [10], [12]. Still in line with our assumption left-hander frequencies among outstanding tennis professionals (i.e. top 10 players) decreased in both men’s and ladies’ tennis. Moreover, as expected there was only an interim excess of left-handed male professionals in top 100 (Fig. 1C) and Grand Slam first round players (Fig. 3A). Even more, logarithmic regressions between ranking intervals and left-hander frequencies support our prediction that performing left-handed in men’s tennis became less relevant for attaining high world ranking over the years (Fig. 2A). Taken together, the findings from tennis professionals indicate that, at least for men’s competition where significantly increased left-hander frequencies were found in between, an enhanced players’ professionalism may reduce and even neutralize the left-handers’ performance advantage over time.

## Study 2: Method

Amateur players lack the professionals’ privilege to have almost unlimited access to match preparation opportunities. We therefore hypothesized to find an increase in left-hander frequencies with higher performance level in a cohort of non-professionals. To this end, we analysed the incidence of left-handed performers registered as players in the German Westphalian Tennis Association (WTV) for the summer season 2008. Data was obtained from an open-source online database (http://wtv.liga.nu/) where each player was assigned a performance level based on his or her performance in the previous season (highest = 1 through lowest = 23). For each of the 597 tennis clubs in the WTV, lists of players were served by post to the clubs’ respective sporting directors or another responsible person. Receivers were asked to complete the lists by adding players’ handedness for racket use in tennis (left- vs. right-handed). A total of 184 clubs responded to our request (Table S7). Ethics approval and informed consent from participants were not obtained for this study since similar to data from tennis professionals the original material (i.e. players’ names, gender, performance level and team) on which our investigation was based was freely available online. In addition, we considered playing handedness to belong to those overt player characteristics that is directly available once one watches an athlete playing tennis. Please note that contacted clubs were free to not return the lists or to keep entries empty in case a player disagreed with the specification of his or her playing handedness in tennis.

### Results and Discussion

**Figure 4:**
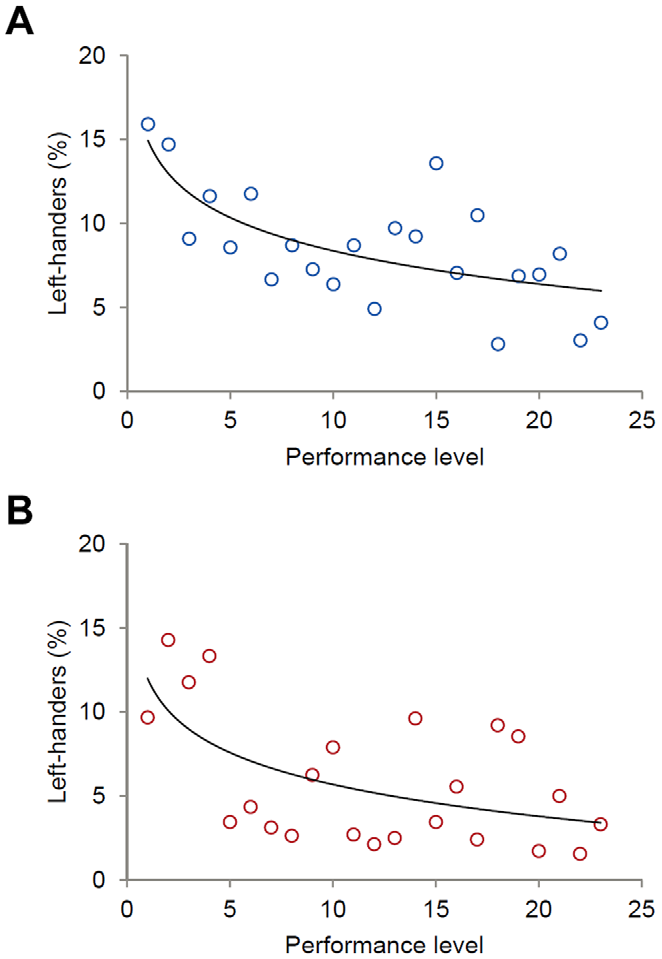
Frequency of left-handed performers in amateur tennis. Left-hander frequencies in (A) male and (B) female amateur tennis depending on performance level (1 =  highest level, …, 23 =  lowest level). Data were fitted to logarithmic functions with *R* ^2^ = .48 (men) and *R* ^2^ = .34 (ladies), respectively (see also Table S7).

Similar to previous findings, left-handed performers were quite rare among players in general [10] and their incidence was higher among males (6.82%, *N* = 2185 players) compared to females (4.42%, *N* = 1608 players) [4]. Importantly, however, for both sexes left-hander frequencies were not equally distributed over performance levels. As expected, frequencies logarithmically increased with higher performance levels in men and women (Fig. 4). Albeit the cross-sectional design limits conclusive interpretation, findings support our hypothesis that a left-handers’ performance advantage persists in amateur tennis.

## General Discussion

Here we significantly furthered our understanding of a potential left-handers’ advantage in tennis by longitudinally tracking handedness frequencies in elite performers and by contrasting our findings with data from non-elite players. Overall, the data suggest that players’ handedness had a moderate (in men) or almost no (in ladies) impact on high achievement in professional tennis [12]. With regard to men’s professionals in particular the trends found in left-hander frequencies over time indicate that playing left-handed did, but no longer does, facilitate the achievement of an exceptional world ranking. Importantly, this is not to say that (elite) tennis players might not feel uncomfortable competing with left-handed opponents today. However, the findings suggest that such discomfort, probably originating from a reduced familiarity with left-handers [3], [11], [12], [15], [26], does no longer result in significantly enhanced proportion of left-handed athletes among top professionals.

We suggest that the steady improvements in match preparation opportunities for tennis professionals play an important role for such development. Today, elite performers and coaches can refer to a myriad of information about future opponents (e.g. by video observation, performance statistics) and use this for an in-depth opponent-specific match preparation. Knowing about a left-handed opponent’s strengths and weaknesses is likely to facilitate the modification of game-play strategies learned through repeated exposure to right-handers, even though these strategies might not be fully switched [17]. Recently, laboratory research confirmed that practicing with left-handers is effective for improving visual anticipation of left-handed action intentions [30], which, in turn, might help players optimally preparing appropriate motor responses to left-handed actions on-court. In contrast, amateur players lack the professionals’ privileges and, as found here, playing tennis left-handed seems to come along with a performance advantage in this cohort.

We followed the strategy applied by [3] to identify if left-handers were significantly overrepresented among the top 100 performers in year-end rankings. We referred to large survey data to obtain observations of handedness distribution in the normal population and we used handedness for throwing because it matches with handedness for holding a racket [28]. By doing so the observed and thus expected left-hander frequencies were 9.98% and 7.5% for men and women, respectively. Other research on handedness in tennis used different reference values (men: 6.98%−12.2%; ladies: 7.69%−10.7%) [10], [12], [29], which has to be taken into account when comparing studies’ results and interpretations of left-hander frequencies as either being significantly different from the normal population or not. Importantly, however, the discrepancy between reference values across studies does not affect our key findings that left-hander frequencies (i) decreased in top 10 performers in men’s and ladies’ tennis during past four decades, (ii) showed an inverse U-shaped pattern in male top 100 players and Grand Slam first round participants and (iii) that left-handedness seems no longer associated with better ranking intervals in men’s year-end world rankings.

Two other aspects deserve closer attention. First, in line with previous research left-handedness was more pronounced in men compared to women in both professional and amateur players [3], [10]. While this effect might be attributed to common gender differences found in the normal population [4], [5], stronger competition in male as opposed to female sport might, among others, also explain why left-handedness occurs more often in male than female athletes [3]. From a sport-specific performance demands perspective, however, the heavier spatiotemporal constraints imposed on players in men’s compared to ladies’ tennis (e.g. greater shot rate in men’s singles) might additionally help unravelling this effect [31].

Second, recent analyses of match data from Grand Slam tennis tournaments in 2005 to 2008 revealed that, once quality differences between players were controlled for, male, but not female, right-handers had about 5.9% lower probability of success against left-handed opponents [32]. Thus, according to that research, left-handed players appeared to have a performance advantage even in modern elite tennis. The discrepancy between these and our findings may be solved by the fact that year-end world rankings, which we have looked at here, are not only composed of the points a player receives from competing at Grand Slam events. In addition, that study considered only a short period of tennis competition (i.e. four years) [32]. The inclusion of match data from the beginning of the open era until today would enable a better assessment of the practical relevance of the 5.9% lower probability of success relative to previous years. Future research is encouraged to meet the challenge to extend the longitudinal approach applied here to other performance measures in tennis or to other interactive sports [18], [19], [33]. Doing so is expected to facilitate our understanding of the relevance of performing left-handed for high sporting achievement just as if and how the potential benefits of left-hand performance interact with athletes’ professionalism.

In summary, our findings support the frequency-dependent advantage hypothesis insofar as the left-handers’ advantage in tennis seems modifiable, probably through the professionals’ more sophisticated match preparation opportunities, extensive domain-specific learning and adaptation processes [24], [25], [30]. With regard to alternative explanations, findings contradict the notion that left-handers in comparison to right-handers might benefit more from long and intense training because of being biologically predisposed [10]. If such naturally determined innate processes were at work and made left-handers superior to right-handers one would expect left-handers to be overrepresented in highly trained tennis professionals even today. Also, to the best of our knowledge, there was no drop in the incidence of left-handers in the normal population over the past forty years or any rule changes in tennis that might explain why the left-handers’ advantage in tennis should have decreased. Looking ahead at the next 40 years of professional tennis we anticipate that the impressive closely spaced success of former left-handed players such as Jimmy Connors, John McEnroe or Martina Navratilova will be difficult to replicate by future left-handed professionals.

## Supporting Information

Table S1 **Handedness in the top 100 year-end rankings in men’s and ladies’ professional tennis.**
(DOCX)

Table S2 **Handedness of players in men’s year-end rankings by ranking interval.**
(DOCX)

Table S3 **Handedness of male professional players in the first rounds of Grand Slam tournaments (1968–2011).**
(DOCX)

Table S4 **Handedness of female professional players in the first rounds of Grand Slam tournaments (1968–2011).**
(DOCX)

Table S5 **Grand Slam finalists in men’s professional tennis (1968–2011).**
(DOCX)

Table S6 **Grand Slam finalists in ladies’ professional tennis (1968–2011).**
(DOCX)

Table S7 **Handedness in men’s and ladies’ amateur tennis across performance levels.**
(DOCX)
