# Supplementary material for: Left-Handedness in Professional and Amateur Tennis
Source: PLoS One. 2012 Nov 7;7(11):e49325. doi: 10.1371/journal.pone.0049325 (PMC3492260; doi:10.1371/journal.pone.0049325)
Supplement: Table S1 — Handedness in the top 100 year-end rankings in men’s and ladies’ professional tennis. (DOCX) [file pone.0049325.s001.docx]

**Table S1. Handedness in the top 100 year-end rankings in men’s and ladies’ professional tennis.**

|  | **Men** | | | | | | **Ladies** | | | | | |
| --- | --- | --- | --- | --- | --- | --- | --- | --- | --- | --- | --- | --- |
| **Year** | **Date (month/day)** | **LH** | **RH** | **AMB** | **N/A** | ***p*** | **Date (month/day)** | **LH** | **RH** | **AMB** | **N/A** | ***p*** |
| 1973 | 12/14 | 13 | 87 | - | - | .196 | - | - | - | - | - | - |
| 1974 | 12/20 | 13 | 87 | - | - | .196 | - | - | - | - | - | - |
| 1975 | 12/15 | 14 | 86 | - | - | .123 | 11/04 | 4 | 81 | - | 15 | .889 |
| 1976 | 12/12 | 14 | 86 | - | - | .123 | 12/05 | 4 | 76 | - | 20 | .859 |
| 1977 | 12/31 | 16 | 84 | - | - | .039 | 12/31 | 7 | 75 | - | 18 | .419 |
| 1978 | 12/31 | 14 | 86 | - | - | .123 | 12/10 | 10 | 79 | - | 11 | .130 |
| 1979 | 12/26 | 19 | 81 | - | - | .005 | 12/31 | 11 | 74 | - | 15 | .053 |
| 1980 | 12/31 | 17 | 83 | - | - | .020 | 12/31 | 10 | 72 | - | 18 | .086 |
| 1981 | 11/23 | 14 | 86 | - | - | .123 | 12/31 | 11 | 72 | - | 17 | .046 |
| 1982 | 12/20 | 16 | 84 | - | - | .039 | 12/20 | 10 | 74 | - | 16 | .098 |
| 1983 | 12/19 | 13 | 85 | 2 | - | .196 | 12/05 | 11 | 75 | - | 14 | .057 |
| 1984 | 12/24 | 16 | 84 | - | - | .039 | 12/24 | 9 | 76 | - | 15 | .186 |
| 1985 | 12/30 | 15 | 84 | 1 | - | .072 | 12/23 | 11 | 81 | - | 8 | .083 |
| 1986 | 12/29 | 13 | 87 | - | - | .196 | 12/22 | 8 | 87 | - | 5 | .420 |
| 1987 | 12/28 | 13 | 87 | - | - | .196 | 12/20 | 9 | 80 | - | 11 | .223 |
| 1988 | 12/19 | 19 | 81 | - | - | .005 | 12/19 | 11 | 81 | - | 8 | .083 |
| 1989 | 12/18 | 15 | 85 | - | - | .072 | 11/20 | 9 | 79 | - | 12 | .214 |
| 1990 | 12/31 | 16 | 84 | - | - | .039 | 11/26 | 8 | 82 | - | 10 | .362 |
| 1991 | 12/30 | 18 | 82 | - | - | .010 | 11/25 | 5 | 87 | - | 8 | .828 |
| 1992 | 12/28 | 21 | 79 | - | - | **.001** | 11/23 | 5 | 87 | - | 8 | .828 |
| 1993 | 12/27 | 17 | 83 | - | - | .020 | 12/27 | 6 | 91 | - | 3 | .743 |
| 1994 | 12/26 | 17 | 83 | - | - | .020 | 11/21 | 5 | 91 | - | 4 | .855 |
| 1995 | 12/25 | 21 | 79 | - | - | **.001** | 12/25 | 3 | 94 | - | 4 | .979 |
| 1996 | 12/30 | 16 | 84 | - | - | .039 | 12/30 | 8 | 87 | - | 5 | .420 |
| 1997 | 12/29 | 13 | 87 | - | - | .196 | 11/24 | 9 | 88 | - | 3 | .303 |
| 1998 | 12/28 | 18 | 82 | - | - | .010 | 11/23 | 11 | 89 | - | - | .129 |
| 1999 | 12/27 | 15 | 85 | - | - | .072 | 12/27 | 10 | 90 | - | - | .217 |
| 2000 | 12/25 | 12 | 88 | - | - | .881 | 12/25 | 7 | 93 | - | - | .630 |
| 2001 | 12/31 | 13 | 87 | - | - | .196 | 11/12 | 9 | 91 | - | - | .335 |
| 2002 | 12/30 | 14 | 86 | - | - | .123 | 11/11 | 9 | 91 | - | - | .335 |
| 2003 | 12/29 | 7 | 93 | - | - | .208 | 11/10 | 11 | 89 | - | - | .129 |
| 2004 | 12/27 | 13 | 87 | - | - | .196 | 12/27 | 5 | 95 | - | - | .878 |
| 2005 | 12/26 | 10 | 90 | - | - | .546 | 11/14 | 8 | 92 | - | - | .478 |
| 2006 | 12/25 | 9 | 91 | - | - | .677 | 12/18 | 8 | 92 | - | - | .478 |
| 2007 | 12/31 | 12 | 88 | - | - | .295 | 11/12 | 7 | 93 | - | - | .630 |
| 2008 | 12/29 | 13 | 87 | - | - | .196 | 11/10 | 8 | 92 | - | - | .478 |
| 2009 | 12/28 | 12 | 88 | - | - | .295 | 11/09 | 8 | 92 | - | - | .478 |
| 2010 | 12/27 | 12 | 88 | - | - | .295 | 11/08 | 10 | 90 | - | - | .217 |
| 2011 | 12/26 | 16 | 84 | - | - | .039 | 11/07 | 8 | 92 | - | - | .478 |

This table shows the handedness distribution (LH = Left-handed player, RH = Right-handed player, AMB = Ambidextrous player, i.e. playing left- and right-handed, N/A = Handedness not available) in the top 100 year-end world rankings for men’s (1973-2011) and ladies’ professional tennis (1975-2011). Date columns list the dates of respective year-end rankings we included in our analyses.

Analogous to the procedure described by Raymond et al. [[1](#_ENREF_1)] *p*-values refer to one-tailed 2 x 2 Fisher exact tests that tested for differences between observed handedness distribution in respective top 100 year-rankings and observed handedness distribution for throwing in the normal population (men: 9.98%, ladies: 7.5%) based on the data reported by Gilbert and Wysocki [[2](#_ENREF_2)] for people aged 18-30 years.

For both sexes, left-handedness for throwing was lower compared to left-handedness for writing (males: 13.03%; females: 10.72%) in the same age group [[2](#_ENREF_2)]. Therefore, in line with previous work [[1](#_ENREF_1)] we ran additional, more conservative, tests by considering handedness for writing (men: *n*_LH_ = 10495, *n*_RH_ = 70029; ladies: *n*_LH_ = 12502, *n*_RH_ = 104161) as the observed handedness distribution in the normal population. Bold *p*-values in the above table indicate significant (*p* < .05) results for these conservative tests. Please note that for the years 1983 and 1985 in men’s rankings, we added the number of ambidextrous players to right-handers so as to not overestimate left-hander frequencies.

**References**

1. Raymond M, Pontier D, Dufour AB, Møller AP (1996) Frequency-dependent maintenance of left handedness in humans. P Roy Soc Lond B Bio 263: 1627-1633.

2. Gilbert AN, Wysocki CJ (1992) Hand preference and age in the United States. Neuropsychologia 30: 601-608.
