# Supplementary material for: Left-Handedness in Professional and Amateur Tennis
Source: PLoS One. 2012 Nov 7;7(11):e49325. doi: 10.1371/journal.pone.0049325 (PMC3492260; doi:10.1371/journal.pone.0049325)
Supplement: Table S2 — Handedness of players in men’s year-end rankings by ranking interval. (DOCX) [file pone.0049325.s002.docx]

**Table S2. Handedness of players in men’s year-end rankings by ranking interval.**

|  |  | **Ranking Interval** | | | | | | | | | |
| --- | --- | --- | --- | --- | --- | --- | --- | --- | --- | --- | --- |
| **Year** | **Hand** | **1** | **2** | **3** | **4** | **5** | **6** | **7** | **8** | **9** | **10** |
| 1973 | LH | 8 | 5 | 3 | 2 | 3 |  |  |  |  |  |
|  | RH | 42 | 45 | 47 | 48 | 32 |  |  |  |  |  |
|  | AMB | - | - | - | - | - |  |  |  |  |  |
| 1974 | LH | 10 | 3 | 5 | 6 | 1 | 2 | 2 | - |  |  |
|  | RH | 40 | 47 | 45 | 44 | 49 | 48 | 48 | 23 |  |  |
|  | AMB | - | - | - | - | - | - | - | - |  |  |
| 1975 | LH | 8 | 6 | 5 | 4 | 2 | 3 | 1 | 1 | - |  |
|  | RH | 42 | 44 | 45 | 46 | 48 | 47 | 49 | 49 | 7 |  |
|  | AMB | - | - | - | - | - | - | - | - | - |  |
| 1976 | LH | 6 | 8 | 6 | 7 | 2 | 2 | 1 | 3 |  |  |
|  | RH | 44 | 42 | 44 | 43 | 48 | 48 | 49 | 38 |  |  |
|  | AMB | - | - | - | - | - | - | - | - |  |  |
| 1977 | LH | 7 | 9 | 8 | 4 | 2 | 2 | 2 | 3 | 1 |  |
|  | RH | 43 | 41 | 42 | 46 | 48 | 48 | 48 | 47 | 49 | 15 |
|  | AMB | - | - | - | - | - | - | - | - | - | - |
| 1978 | LH | 8 | 6 | 5 | 4 | 3 | 4 | 3 | 2 | 1 | 1 |
|  | RH | 42 | 44 | 45 | 46 | 47 | 46 | 47 | 48 | 49 | 49 |
|  | AMB | - | - | - | - | - | - | - | - | - | - |
| 1979 | LH | 7 | 12 | 6 | 3 | 1 | 2 | 6 | 1 | 2 | 1 |
|  | RH | 43 | 38 | 44 | 47 | 49 | 48 | 44 | 48 | 47 | 49 |
|  | AMB | - | - | - | - | - | - | - | 1 | 1 | - |
| 1980 | LH | 6 | 11 | 7 | - | 4 | 3 | 7 | 5 | 3 | 1 |
|  | RH | 44 | 39 | 43 | 49 | 46 | 47 | 43 | 44 | 47 | 49 |
|  | AMB | - | - | - | 1 | - | - | - | 1 | - | - |
| 1981 | LH | 8 | 6 | 6 | 5 | 11 | 5 | 1 |  |  |  |
|  | RH | 42 | 44 | 43 | 44 | 39 | 45 | - |  |  |  |
|  | AMB | - | - | 1 | 1 | - | - | - |  |  |  |
| 1982 | LH | 8 | 8 | 8 | 3 | 7 | 5 | 6 | 1 | 2 | 2 |
|  | RH | 42 | 42 | 42 | 46 | 43 | 45 | 44 | 49 | 48 | 48 |
|  | AMB | - | - | - | 1 | - | - | - | - | - | - |
| 1983 | LH | 6 | 7 | 8 | 6 | 10 | 2 | 7 | 5 | 1 | 5 |
|  | RH | 43 | 42 | 42 | 44 | 40 | 48 | 43 | 45 | 49 | 45 |
|  | AMB | 1 | 1 | - | - | - | - | - | - | - | - |
| 1984 | LH | 9 | 7 | 5 | 5 | 7 | 9 | 6 | 7 | 3 | 5 |
|  | RH | 41 | 43 | 44 | 45 | 43 | 40 | 44 | 43 | 47 | 45 |
|  | AMB | - | - | 1 | - | - | 1 | - | - | - | - |
| 1985 | LH | 7 | 8 | 9 | 7 | 7 | - | 7 | 7 | 5 | 4 |
|  | RH | 43 | 41 | 41 | 43 | 42 | 50 | 43 | 43 | 44 | 46 |
|  | AMB | - | 1 | - | - | 1 | - | - | - | 1 | - |
| 1986 | LH | 9 | 4 | 10 | 7 | 6 | 4 | 8 | 4 | 3 | 2 |
|  | RH | 41 | 46 | 39 | 43 | 43 | 46 | 42 | 46 | 46 | 48 |
|  | AMB | - | - | 1 | - | 1 | - | - | - | 1 | - |
| 1987 | LH | 5 | 8 | 10 | 9 | 6 | 4 | 5 | 10 | 5 | 4 |
|  | RH | 45 | 42 | 40 | 38 | 44 | 46 | 45 | 40 | 45 | 46 |
|  | AMB | - | - | - | 3 | - | - | - | - | - | - |
| 1988 | LH | 7 | 12 | 9 | 6 | 6 | 5 | 5 | 6 | 5 | 5 |
|  | RH | 43 | 38 | 41 | 42 | 44 | 44 | 45 | 44 | 45 | 45 |
|  | AMB | - | - | - | 2 | - | 1 | - | - | - | - |
| 1989 | LH | 8 | 7 | 12 | 9 | 8 | 3 | 5 | 7 | 9 | 3 |
|  | RH | 42 | 43 | 38 | 40 | 42 | 47 | 44 | 43 | 41 | 46 |
|  | AMB | - | - | - | 1 | - | - | 1 | - | - | 1 |
| 1990 | LH | 10 | 6 | 11 | 13 | 3 | 4 | 5 | 10 | 5 | 4 |
|  | RH | 40 | 44 | 39 | 37 | 46 | 46 | 45 | 40 | 44 | 46 |
|  | AMB | - | - | - | - | 1 | - | - | - | 1 | - |
| 1991 | LH | 10 | 8 | 12 | 10 | 8 | 4 | 3 | 2 | 4 | 3 |
|  | RH | 40 | 42 | 38 | 40 | 42 | 46 | 47 | 48 | 46 | 47 |
|  | AMB | - | - | - | - | - | - | - | - | - | - |
| 1992 | LH | 11 | 10 | 9 | 8 | 5 | 6 | 9 | 6 | 4 | 6 |
|  | RH | 39 | 40 | 41 | 42 | 45 | 44 | 41 | 44 | 46 | 44 |
|  | AMB | - | - | - | - | - | - | - | - | - | - |
| 1993 | LH | 7 | 10 | 9 | 8 | 6 | 4 | 9 | 11 | 3 | 5 |
|  | RH | 43 | 40 | 41 | 42 | 44 | 46 | 41 | 39 | 47 | 45 |
|  | AMB | - | - | - | - | - | - | - | - | - | - |
| 1994 | LH | 7 | 10 | 8 | 7 | 11 | 4 | 7 | 5 | 6 | 7 |
|  | RH | 43 | 40 | 42 | 43 | 39 | 46 | 43 | 45 | 44 | 43 |
|  | AMB | - | - | - | - | - | - | - | - | - | - |
| 1995 | LH | 8 | 13 | 6 | 7 | 4 | 7 | 7 | 7 | 7 | 2 |
|  | RH | 42 | 37 | 44 | 43 | 46 | 43 | 43 | 42 | 43 | 48 |
|  | AMB | - | - | - | - | - | - | - | 1 | - | - |
| 1996 | LH | 8 | 8 | 10 | 4 | 7 | 10 | 5 | 4 | 3 | 4 |
|  | RH | 42 | 42 | 40 | 46 | 43 | 40 | 44 | 46 | 47 | 46 |
|  | AMB | - | - | - | - | - | - | 1 | - | - | - |
| 1997 | LH | 9 | 4 | 12 | 12 | 7 | 3 | 3 | 3 | 4 | 6 |
|  | RH | 41 | 46 | 38 | 38 | 43 | 47 | 47 | 47 | 46 | 45 |
|  | AMB | - | - | - | - | - | - | - | - | - | - |
| 1998 | LH | 10 | 8 | 7 | 5 | 4 | 7 | 6 | 4 | 1 | 2 |
|  | RH | 40 | 42 | 43 | 45 | 46 | 43 | 44 | 46 | 49 | 48 |
|  | AMB | - | - | - | - | - | - | - | - | - | - |
| 1999 | LH | 7 | 8 | 4 | 5 | 8 | 10 | 4 | 2 | 5 | 6 |
|  | RH | 43 | 42 | 46 | 45 | 42 | 40 | 46 | 48 | 45 | 44 |
|  | AMB | - | - | - | - | - | - | - | - | - | - |
| 2000 | LH | 7 | 5 | 8 | 7 | 8 | 4 | 8 | 4 | 6 | 2 |
|  | RH | 43 | 45 | 42 | 43 | 42 | 46 | 42 | 46 | 44 | 48 |
|  | AMB | - | - | - | - | - | - | - | - | - | - |
| 2001 | LH | 6 | 7 | 5 | 10 | 10 | 4 | 4 | 4 | 6 | 5 |
|  | RH | 44 | 43 | 45 | 40 | 40 | 46 | 46 | 46 | 44 | 45 |
|  | AMB | - | - | - | - | - | - | - | - | - | - |
| 2002 | LH | 3 | 11 | 6 | 6 | 6 | 10 | 2 | 10 | 4 | 7 |
|  | RH | 47 | 39 | 44 | 44 | 44 | 40 | 48 | 40 | 45 | 43 |
|  | AMB | - | - | - | - | - | - | - | - | 1 | - |
| 2003 | LH | 3 | 4 | 13 | 9 | 3 | 4 | 8 | 8 | 6 | 6 |
|  | RH | 47 | 46 | 37 | 41 | 47 | 46 | 42 | 42 | 44 | 44 |
|  | AMB | - | - | - | - | - | - | - | - | - | - |
| 2004 | LH | 5 | 8 | 5 | 8 | 4 | 6 | 9 | 7 | 6 | 9 |
|  | RH | 45 | 42 | 45 | 42 | 46 | 44 | 41 | 43 | 44 | 40 |
|  | AMB | - | - | - | - | - | - | - | - | - | 1 |
| 2005 | LH | 5 | 5 | 6 | 5 | 8 | 6 | 5 | 7 | 6 | 9 |
|  | RH | 45 | 45 | 44 | 45 | 42 | 44 | 45 | 43 | 43 | 41 |
|  | AMB | - | - | - | - | - | - | - | - | 1 | - |
| 2006 | LH | 4 | 5 | 8 | 9 | 3 | 4 | 8 | 7 | 6 | 8 |
|  | RH | 46 | 45 | 42 | 41 | 47 | 46 | 41 | 42 | 44 | 42 |
|  | AMB | - | - | - | - | - | - | 1 | 1 | - | - |
| 2007 | LH | 5 | 7 | 6 | 7 | 7 | 4 | 5 | 11 | 8 | 6 |
|  | RH | 45 | 43 | 44 | 43 | 43 | 45 | 45 | 39 | 42 | 44 |
|  | AMB | - | - | - | - | - | 1 | - | - | - | - |
| 2008 | LH | 6 | 7 | 8 | 10 | 2 | 4 | 6 | 11 | 5 | 6 |
|  | RH | 44 | 43 | 42 | 40 | 48 | 46 | 43 | 39 | 44 | 43 |
|  | AMB | - | - | - | - | - | - | 1 | - | 1 | 1 |
| 2009 | LH | 7 | 5 | 6 | 10 | 13 | 1 | 5 | 10 | 6 | 6 |
|  | RH | 43 | 45 | 44 | 40 | 37 | 49 | 44 | 40 | 42 | 44 |
|  | AMB | - | - | - | - | - | - | 1 | - | 2 | - |
| 2010 | LH | 7 | 5 | 9 | 9 | 7 | 6 | 6 | 7 | 6 | 8 |
|  | RH | 43 | 45 | 41 | 41 | 43 | 44 | 44 | 43 | 42 | 41 |
|  | AMB | - | - | - | - | - | - | - | - | 2 | 1 |
| 2011 | LH | 7 | 9 | 9 | 9 | 6 | 6 | 5 | 6 | 6 | 3 |
|  | RH | 43 | 41 | 41 | 41 | 44 | 44 | 45 | 44 | 43 | 47 |
|  | AMB | - | - | - | - | - | - | - | - | 1 | - |

This table lists the handedness distribution (LH = Left-handed player, RH = Right-handed player, AMB = Ambidextrous player, i.e. playing left- and right-handed) found in ranking intervals of 50 players (i.e. 1 = top 50 players, 2 = 51-100, …, 10 = 451-500) in men’s year-end world rankings from 1973-2011. Left-hander frequencies (in %) were fitted to ranking intervals using logarithmic functions. Ranking intervals that included less than 20 players were not included in those fittings.

For the sake of completeness, we also calculated linear and quadratic fittings and compared the squared residuals obtained from each of the three fitting procedures for each year-end ranking by means of Friedman tests. Significant differences between fitting procedures were found for the years 1973 (χ²(2, *N* = 5) = 8.40, *p* = .02) and 1982 (χ²(2, *N* = 10) = 9.80, *p* < .01). For 1973, a quadratic fitting performed best (*M* = 0.48, *SD* = 0.52; linear: *M* = 8.13, *SD* = 4.84; logarithmic: *M* = 4.91, *SD* = 5.00), while for 1982, both linear (*M* = 9.36, *SD* = 12.66) and quadratic fittings (*M* = 9.31, *SD* = 13.22) yielded similar, however, better performance than the logarithmic fitting (*M* = 11.67, *SD* = 9.05).
