# Supplementary material for: Left-Handedness in Professional and Amateur Tennis
Source: PLoS One. 2012 Nov 7;7(11):e49325. doi: 10.1371/journal.pone.0049325 (PMC3492260; doi:10.1371/journal.pone.0049325)
Supplement: Table S3 — Handedness of male professional players in the first rounds of Grand Slam tournaments (1968–2011). (DOCX) [file pone.0049325.s003.docx]

**Table S3. Handedness of male professional players in the first rounds of Grand Slam tournaments (1968-2011).**

|  | **Australian Open** | | | | **French Open** | | | | **Wimbledon** | | | | **US Open** | | | |
| --- | --- | --- | --- | --- | --- | --- | --- | --- | --- | --- | --- | --- | --- | --- | --- | --- |
|  | **LH** | **RH** | **AMB** | **N/A** | **LH** | **RH** | **AMB** | **N/A** | **LH** | **RH** | **AMB** | **N/A** | **LH** | **RH** | **AMB** | **N/A** |
| 1968 | 2 | 60 | - | - | 9 | 118 | - | 1 | 13 | 115 | - | - | 8 | 88 | - | - |
| 1969 | 3 | 45 | - | - | 9 | 119 | - | - | 14 | 114 | - | - | 11 | 116 | - | 1 |
| 1970 | 4 | 44 | - | - | 6 | 122 | - | - | 14 | 114 | - | - | 10 | 98 | - | - |
| 1971 | 8 | 40 | - | - | 10 | 118 | - | - | 15 | 113 | - | - | 12 | 116 | - | - |
| 1972 | 6 | 43 | - | - | 6 | 58 | - | - | 13 | 114 | - | 1 | 16 | 112 | - | - |
| 1973 | 4 | 52 | - | - | 13 | 115 | - | - | 12 | 116 | - | - | 16 | 112 | - | - |
| 1974 | 7 | 57 | - | - | 11 | 117 | - | - | 18 | 110 | - | - | 18 | 110 | - | - |
| 1975 | 3 | 29 | - | - | 13 | 115 | - | - | 17 | 111 | - | - | 14 | 114 | - | - |
| 1976 | 3 | 61 | - | - | 15 | 113 | - | - | 15 | 113 | - | - | 16 | 112 | - | - |
| 1977 * | 8 | 56 | - | - | 16 | 112 | - | - | 16 | 112 | - | - | 20 | 108 | - | - |
|  | 6 | 58 | - | - |  |  |  |  |  |  |  |  |  |  |  |  |
| 1978 | 8 | 56 | - | - | 18 | 110 | - | - | 18 | 110 | - | - | 18 | 110 | - | - |
| 1979 | 7 | 57 | - | - | 16 | 112 | - | - | 18 | 110 | - | - | 18 | 110 | - | - |
| 1980 | 8 | 56 | - | - | 17 | 111 | - | - | 19 | 109 | - | - | 20 | 108 | - | - |
| 1981 | 8 | 56 | - | - | 15 | 113 | - | - | 15 | 112 | 1 | - | 19 | 108 | 1 | - |
| 1982 | 15 | 81 | - | - | 19 | 109 | - | - | 19 | 109 | - | - | 23 | 105 | - | - |
| 1983 | 14 | 82 | - | - | 16 | 112 | - | - | 19 | 109 | - | - | 22 | 106 | - | - |
| 1984 | 10 | 86 | - | - | 17 | 109 | 2 | - | 18 | 109 | 1 | - | 20 | 105 | 1 | - |
| 1985 | 19 | 77 | - | - | 18 | 109 | 1 | - | 20 | 108 | - | - | 19 | 107 | 2 | - |
| 1986 | no competition | | | | 18 | 109 | 1 | - | 20 | 108 | - | - | 19 | 108 | 1 | - |
| 1987 | 18 | 78 | - | - | 17 | 110 | 1 | - | 18 | 109 | 1 | - | 20 | 108 | - | - |
| 1988 | 22 | 106 | - | - | 19 | 109 | - | - | 21 | 107 | - | - | 23 | 105 | - | - |
| 1989 | 24 | 104 | - | - | 14 | 114 | - | - | 19 | 109 | - | - | 22 | 106 | - | - |
| 1990 | 24 | 104 | - | - | 20 | 108 | - | - | 22 | 106 | - | - | 23 | 105 | - | - |
| 1991 | 21 | 107 | - | - | 25 | 103 | - | - | 27 | 101 | - | - | 25 | 103 | - | - |
| 1992 | 27 | 101 | - | - | 30 | 98 | - | - | 27 | 101 | - | - | 25 | 103 | - | - |
| 1993 | 22 | 106 | - | - | 22 | 106 | - | - | 25 | 103 | - | - | 24 | 104 | - | - |
| 1994 | 22 | 106 | - | - | 20 | 108 | - | - | 23 | 105 | - | - | 23 | 105 | - | - |
| 1995 | 22 | 105 | 1 | - | 25 | 103 | - | - | 26 | 102 | - | - | 24 | 103 | 1 | - |
| 1996 | 25 | 103 | - | - | 23 | 105 | - | - | 19 | 109 | - | - | 21 | 107 | - | - |
| 1997 | 19 | 109 | - | - | 25 | 103 | - | - | 27 | 101 | - | - | 19 | 109 | - | - |
| 1998 | 21 | 107 | - | - | 20 | 108 | - | - | 21 | 107 | - | - | 22 | 105 | - | 1 |
| 1999 | 21 | 107 | - | - | 21 | 107 | - | - | 18 | 110 | - | - | 19 | 109 | - | - |
| 2000 | 17 | 111 | - | - | 18 | 110 | - | - | 22 | 106 | - | - | 20 | 108 | - | - |
| 2001 | 15 | 113 | - | - | 17 | 111 | - | - | 22 | 106 | - | - | 16 | 112 | - | - |
| 2002 | 17 | 111 | - | - | 14 | 114 | - | - | 22 | 106 | - | - | 19 | 109 | - | - |
| 2003 | 18 | 110 | - | - | 17 | 111 | - | - | 20 | 108 | - | - | 16 | 112 | - | - |
| 2004 | 16 | 112 | - | - | 13 | 115 | - | - | 16 | 112 | - | - | 17 | 111 | - | - |
| 2005 | 14 | 114 | - | - | 16 | 112 | - | - | 17 | 111 | - | - | 15 | 113 | - | - |
| 2006 | 13 | 115 | - | - | 13 | 115 | - | - | 16 | 112 | - | - | 13 | 115 | - | - |
| 2007 | 15 | 113 | - | - | 13 | 115 | - | - | 15 | 113 | - | - | 14 | 114 | - | - |
| 2008 | 16 | 112 | - | - | 20 | 108 | - | - | 20 | 108 | - | - | 20 | 107 | - | 1 |
| 2009 | 20 | 108 | - | - | 16 | 112 | - | - | 16 | 114 | - | - | 17 | 111 | - | - |
| 2010 | 15 | 113 | - | - | 14 | 114 | - | - | 15 | 113 | - | - | 19 | 109 | - | - |
| 2011 | 19 | 109 | - | - | 18 | 110 | - | - | 19 | 109 | - | - | 17 | 111 | - | - |

This table lists the number of male first round players whose handedness for playing tennis was known (LH = Left-handed player, RH = Right-handed player, AMB = Ambidextrous player, i.e. playing left- and right-handed) or unknown (N/A = handedness not available) for each Grand Slam tournament and for each year.

* The Australian Open were carried out twice in 1977 (January and December).
