# Supplementary material for: Left-Handedness in Professional and Amateur Tennis
Source: PLoS One. 2012 Nov 7;7(11):e49325. doi: 10.1371/journal.pone.0049325 (PMC3492260; doi:10.1371/journal.pone.0049325)
Supplement: Table S4 — Handedness of female professional players in the first rounds of Grand Slam tournaments (1968–2011). (DOCX) [file pone.0049325.s004.docx]

**Table S4. Handedness of female professional players in the first rounds of Grand Slam tournaments (1968-2011).**

|  | **Australian Open** | | | | | **French Open** | | | | | **Wimbledon** | | | | | **US Open** | | | |
| --- | --- | --- | --- | --- | --- | --- | --- | --- | --- | --- | --- | --- | --- | --- | --- | --- | --- | --- | --- |
|  | **RH** | **LH** | **AMB** | **N/A** | **RH** | | **LH** | **AMB** | **N/A** | **RH** | | **LH** | **AMB** | **N/A** | **RH** | | **LH** | **AMB** | **N/A** |
| 1968 | 18 | - | 1 | 43 | 37 | | 1 | - | 58 | 51 | | 2 | 1 | 42 | 29 | | 2 | - | 33 |
| 1969 | 12 | 1 | 1 | 18 | 34 | | 3 | 1 | 26 | 48 | | 2 | 2 | 44 | 41 | | 2 | - | 21 |
| 1970 | 12 | - | 1 | 30 | 38 | | 1 | 2 | 21 | 51 | | 3 | 2 | 40 | 35 | | 2 | 1 | 26 |
| 1971 | 12 | 1 | - | 17 | 35 | | 3 | 2 | 24 | 51 | | 4 | 2 | 39 | 41 | | 3 | 1 | 19 |
| 1972 | 9 | - | - | 23 | 35 | | 2 | 2 | 17 | 57 | | 4 | 2 | 33 | 44 | | 2 | 1 | 17 |
| 1973 | 14 | 1 | - | 33 | 37 | | 3 | 2 | 22 | 61 | | 6 | 2 | 27 | 46 | | 2 | - | 16 |
| 1974 | 22 | 1 | - | 33 | 32 | | 4 | 2 | 26 | 65 | | 4 | 1 | 26 | 48 | | 4 | - | 12 |
| 1975 | 21 | 2 | - | 33 | 41 | | 4 | 2 | 17 | 68 | | 4 | 1 | 23 | 57 | | 3 | - | 4 |
| 1976 | 16 | - | - | 16 | 44 | | 1 | - | 19 | 67 | | 3 | - | 26 | 76 | | 4 | - | 16 |
| 1977 * | 14 | 1 | - | 17 | 48 | | 2 | - | 14 | 70 | | 5 | - | 21 | 75 | | 7 | - | 15 |
|  | 10 | - | - | 22 |  | |  |  |  |  | |  |  |  |  | |  |  |  |
| 1978 | 15 | - | - | 17 | 50 | | 3 | - | 11 | 73 | | 7 | - | 13 | 75 | | 10 | - | 11 |
| 1979 | 9 | - | - | 23 | 53 | | 4 | - | 7 | 76 | | 9 | - | 11 | 76 | | 10 | - | 10 |
| 1980 | 35 | 3 | - | 18 | 51 | | 6 | - | 7 | 67 | | 8 | - | 21 | 72 | | 8 | - | 16 |
| 1981 | 43 | 4 | - | 9 | 71 | | 7 | - | 18 | 68 | | 9 | - | 19 | 87 | | 11 | - | 30 |
| 1982 | 45 | 7 | - | 4 | 71 | | 6 | - | 19 | 74 | | 8 | - | 14 | 87 | | 11 | - | 30 |
| 1983 | 46 | 7 | - | 11 | 93 | | 10 | - | 25 | 85 | | 13 | - | 30 | 96 | | 12 | - | 20 |
| 1984 | 49 | 8 | - | 7 | 93 | | 8 | - | 27 | 91 | | 13 | - | 24 | 95 | | 11 | - | 22 |
| 1985 | 44 | 9 | - | 11 | 84 | | 15 | - | 29 | 93 | | 11 | - | 24 | 101 | | 14 | - | 13 |
| 1986 | no competition | | | | 96 | | 12 | - | 20 | 100 | | 13 | - | 15 | 101 | | 13 | - | 14 |
| 1987 | 61 | 11 | - | 24 | 96 | | 12 | - | 20 | 102 | | 13 | - | 13 | 108 | | 12 | - | 8 |
| 1988 | 78 | 8 | - | 42 | 96 | | 10 | - | 22 | 96 | | 15 | - | 17 | 101 | | 15 | - | 12 |
| 1989 | 83 | 13 | - | 32 | 94 | | 12 | - | 22 | 97 | | 13 | - | 18 | 102 | | 14 | - | 12 |
| 1990 | 101 | 10 | - | 17 | 96 | | 14 | - | 18 | 105 | | 12 | - | 11 | 106 | | 14 | - | 8 |
| 1991 | 96 | 11 | - | 21 | 102 | | 10 | - | 16 | 107 | | 8 | - | 13 | 106 | | 12 | - | 10 |
| 1992 | 105 | 12 | - | 11 | 105 | | 10 | - | 13 | 101 | | 12 | - | 15 | 108 | | 12 | - | 8 |
| 1993 | 102 | 12 | - | 14 | 109 | | 10 | - | 9 | 108 | | 10 | - | 10 | 116 | | 7 | - | 5 |
| 1994 | 114 | 6 | - | 8 | 118 | | 5 | - | 5 | 111 | | 9 | - | 8 | 117 | | 7 | - | 4 |
| 1995 | 116 | 4 | - | 8 | 114 | | 6 | - | 8 | 113 | | 6 | - | 9 | 118 | | 5 | - | 5 |
| 1996 | 114 | 8 | - | 6 | 117 | | 10 | - | 1 | 114 | | 10 | - | 4 | 118 | | 8 | - | 2 |
| 1997 | 114 | 9 | - | 5 | 113 | | 12 | - | 3 | 112 | | 13 | - | 3 | 116 | | 10 | - | 2 |
| 1998 | 112 | 11 | - | 5 | 112 | | 12 | - | 4 | 113 | | 13 | - | 2 | 114 | | 11 | - | 3 |
| 1999 | 115 | 12 | - | 1 | 115 | | 13 | - | - | 113 | | 15 | - | - | 115 | | 13 | - | - |
| 2000 | 114 | 14 | - | - | 114 | | 13 | - | 1 | 115 | | 13 | - | - | 116 | | 12 | - | - |
| 2001 | 116 | 12 | - | - | 119 | | 9 | - | - | 115 | | 13 | - | - | 115 | | 13 | - | - |
| 2002 | 112 | 16 | - | - | 114 | | 14 | - | - | 114 | | 14 | - | - | 114 | | 14 | - | - |
| 2003 | 113 | 15 | - | - | 115 | | 13 | - | - | 115 | | 13 | - | - | 114 | | 14 | - | - |
| 2004 | 116 | 12 | - | - | 116 | | 12 | - | - | 117 | | 11 | - | - | 118 | | 10 | - | - |
| 2005 | 117 | 11 | - | - | 120 | | 8 | - | - | 117 | | 11 | - | - | 119 | | 9 | - | - |
| 2006 | 118 | 10 | - | - | 118 | | 10 | - | - | 119 | | 9 | - | - | 117 | | 11 | - | - |
| 2007 | 117 | 11 | - | - | 117 | | 11 | - | - | 117 | | 11 | - | - | 119 | | 9 | - | - |
| 2008 | 118 | 10 | - | - | 115 | | 13 | - | - | 116 | | 12 | - | - | 118 | | 10 | - | - |
| 2009 | 118 | 10 | - | - | 117 | | 11 | - | - | 118 | | 9 | 1 | - | 116 | | 12 | - | - |
| 2010 | 116 | 11 | 1 | - | 115 | | 13 | - | - | 116 | | 12 | - | - | 118 | | 10 | - | - |
| 2011 | 117 | 11 | - | - | 116 | | 12 | - | - | 115 | | 13 | - | - | 116 | | 12 | - | - |

This table lists the number of female first round players whose handedness for playing tennis was known (LH = Left-handed player, RH = Right-handed player, AMB = Ambidextrous player, i.e. playing left- and right-handed) or unknown (N/A = handedness not available) for each Grand Slam tournament and for each year.

* The Australian Open were carried out twice in 1977 (January and December).
