# Supplementary material for: Left-Handedness in Professional and Amateur Tennis
Source: PLoS One. 2012 Nov 7;7(11):e49325. doi: 10.1371/journal.pone.0049325 (PMC3492260; doi:10.1371/journal.pone.0049325)
Supplement: Table S5 — Grand Slam finalists in men’s professional tennis (1968–2011). (DOCX) [file pone.0049325.s005.docx]

**Table S5. Grand Slam finalists in men’s professional tennis (1968-2011).**

| **Player** | **Hand** | **Winner** | **Runner-up** | **First** | **Last** |
| --- | --- | --- | --- | --- | --- |
| Roger Federer | RH | 16 | 7 | 2003 | 2011 |
| Pete Sampras | RH | 14 | 4 | 1990 | 2002 |
| Bjorn Borg | RH | 11 | 5 | 1974 | 1981 |
| Rafael Nadal | LH | 10 | 4 | 2005 | 2011 |
| Ivan Lendl | RH | 8 | 11 | 1981 | 1991 |
| Andre Agassi | RH | 8 | 7 | 1990 | 2005 |
| Jimmy Connors | LH | 8 | 7 | 1974 | 1984 |
| John McEnroe | LH | 7 | 4 | 1979 | 1985 |
| Mats Wilander | RH | 7 | 4 | 1982 | 1988 |
| Stefan Edberg | RH | 6 | 5 | 1985 | 1993 |
| Boris Becker | RH | 6 | 4 | 1985 | 1996 |
| John Newcombe | RH | 5 | 2 | 1969 | 1976 |
| Rod Laver | LH | 5 | 1 | 1968 | 1969 |
| Guillermo Vilas | LH | 4 | 4 | 1975 | 1982 |
| Ken Rosewall | RH | 4 | 4 | 1968 | 1974 |
| Jim Courier | RH | 4 | 3 | 1991 | 1993 |
| Novak Djokovic | RH | 4 | 2 | 2007 | 2011 |
| Arthur Ashe | RH | 3 | 2 | 1968 | 1975 |
| Jan Kodes | RH | 3 | 2 | 1970 | 1973 |
| Gustavo Kuerten | RH | 3 | - | 1997 | 2001 |
| Ilie Nastase | RH | 2 | 3 | 1971 | 1976 |
| Lleyton Hewitt | RH | 2 | 2 | 2001 | 2005 |
| Marat Safin | RH | 2 | 2 | 2000 | 2005 |
| Patrick Rafter | RH | 2 | 2 | 1997 | 2001 |
| Sergi Bruguera | RH | 2 | 1 | 1993 | 1997 |
| Stan Smith | RH | 2 | 1 | 1971 | 1972 |
| Yevgeny Kafelnikov | RH | 2 | 1 | 1996 | 2000 |
| Johan Kriek | RH | 2 | - | 1981 | 1982 |
| Andy Roddick | RH | 1 | 4 | 2003 | 2009 |
| Goran Ivanisevic | LH | 1 | 3 | 1992 | 2001 |
| Michael Chang | RH | 1 | 3 | 1989 | 1996 |
| Juan Carlos Ferrero | RH | 1 | 2 | 2002 | 2003 |
| Michael Stich | RH | 1 | 2 | 1991 | 1996 |
| Pat Cash | RH | 1 | 2 | 1987 | 1988 |
| Vitas Gerulaitis | RH | 1 | 2 | 1977 | 1980 |
| Andres Gimeno | RH | 1 | 1 | 1969 | 1972 |
| Carlos Moya | RH | 1 | 1 | 1997 | 1998 |
| Manuel Orantes | LH | 1 | 1 | 1974 | 1975 |
| Petr Korda | LH | 1 | 1 | 1992 | 1998 |
| Roscoe Tanner | LH | 1 | 1 | 1977 | 1979 |
| Adriano Panatta | RH | 1 | - | 1976 | - |
| Albert Costa | RH | 1 | - | 2002 | - |
| Andres Gomez | LH | 1 | - | 1990 | - |
| Bill Bowrey | RH | 1 | - | 1968 | - |
| Brian Teacher | RH | 1 | - | 1980 | - |
| Gaston Gaudio | RH | 1 | - | 2004 | - |
| Juan Martin Del Potro | RH | 1 | - | 2009 | - |
| Mark Edmondson | RH | 1 | - | 1976 | - |
| Richard Krajicek | RH | 1 | - | 1996 | - |
| Thomas Johannson | RH | 1 | - | 2002 | - |
| Thomas Muster | LH | 1 | - | 1995 | - |
| Yannick Noah | RH | 1 | - | 1983 | - |
| Andy Murray | RH | - | 3 | 2008 | 2011 |
| Tony Roche | LH | - | 3 | 1968 | 1970 |
| Alex Corretja | RH | - | 2 | 1998 | 2001 |
| Cedric Pioline | RH | - | 2 | 1993 | 1997 |
| Kevin Curren | RH | - | 2 | 1984 | 1985 |
| Mark Philippoussis | RH | - | 2 | 1998 | 2003 |
| Miloslav Mecir | RH | - | 2 | 1986 | 1989 |
| Robin Soderling | RH | - | 2 | 2009 | 2010 |
| Steve Denton | RH | - | 2 | 1981 | 1982 |
| Todd Martin | RH | - | 2 | 1994 | 1999 |
| Alberto Berasategui | RH | - | 1 | 1994 | - |
| Alex Metreveli | RH | - | 1 | 1973 | - |
| Andre Medvedev | RH | - | 1 | 1999 | - |
| Arnaud Clement | RH | - | 1 | 2001 | - |
| Brian Gottfried | RH | - | 1 | 1977 | - |
| Chris Lewis | RH | - | 1 | 1983 | - |
| David Nalbandian | RH | - | 1 | 2002 | - |
| Dick Crealy | RH | - | 1 | 1970 | - |
| Fernando Gonzalez | RH | - | 1 | 2007 | - |
| Greg Rusedski | LH | - | 1 | 1997 | - |
| Guillermo Coria | RH | - | 1 | 2004 | - |
| Harold Soloman | RH | - | 1 | 1976 | - |
| Henri Leconte | LH | - | 1 | 1988 | - |
| John Lloyd | RH | - | 1 | 1977 | - |
| John Marks | RH | - | 1 | 1978 | - |
| John Sadri | RH | - | 1 | 1979 | - |
| Jo-Wilfried Tsonga | RH | - | 1 | 2008 | - |
| Juan Gisbert | RH | - | 1 | 1968 | - |
| Kim Warwick | RH | - | 1 | 1980 | - |
| Magnus Norman | RH | - | 1 | 2000 | - |
| Mal Anderson | RH | - | 1 | 1972 | - |
| MaliVai Washington | RH | - | 1 | 1996 | - |
| Marcelo Rios | LH | - | 1 | 1998 | - |
| Marcos Baghdatis | RH | - | 1 | 2006 | - |
| Mariano Puerta | LH | - | 1 | 2005 | - |
| Martin Verkerk | RH | - | 1 | 2003 | - |
| Mikael Pernfors | RH | - | 1 | 1986 | - |
| Nikola Pilic | LH | - | 1 | 1973 | - |
| Onny Parun | RH | - | 1 | 1973 | - |
| Patrick Proisy | RH | - | 1 | 1972 | - |
| Phil Dent | RH | - | 1 | 1974 | - |
| Rainer Schuettler | RH | - | 1 | 2003 | - |
| Thomas Enqvist | RH | - | 1 | 1999 | - |
| Tom Okker | RH | - | 1 | 1968 | - |
| Tomas Berdych | RH | - | 1 | 2010 | - |
| Victor Pecci | RH | - | 1 | 1979 | - |
| Zeljiko Franulovic | RH | - | 1 | 1970 | - |

For each male player who ever made it into a Grand Slam final in the open era (1968-2011) the table lists the full name, the hand used for playing tennis (LH = Left-handed player, RH = Right-handed player), the number of finals won and lost, and the first and the last year (only for players with more than one final played) a player was a Grand Slam finalist.
