# Supplementary material for: Left-Handedness in Professional and Amateur Tennis
Source: PLoS One. 2012 Nov 7;7(11):e49325. doi: 10.1371/journal.pone.0049325 (PMC3492260; doi:10.1371/journal.pone.0049325)
Supplement: Table S6 — Grand Slam finalists in ladies’ professional tennis (1968–2011). (DOCX) [file pone.0049325.s006.docx]

**Table S6. Grand Slam finalists in ladies’ professional tennis (1968-2011).**

| **Player** | **Hand** | **Winner** | **Runner-up** | **First** | **Last** |
| --- | --- | --- | --- | --- | --- |
| Steffi Graf | RH | 22 | 9 | 1987 | 1999 |
| Chris Evert | RH | 18 | 16 | 1973 | 1988 |
| Martina Navratilova | LH | 18 | 14 | 1975 | 1994 |
| Serena Williams | RH | 13 | 4 | 1999 | 2011 |
| Margaret Smith-Court | RH | 12 | 2 | 1968 | 1973 |
| Billie Jean King | RH | 9 | 4 | 1968 | 1975 |
| Monica Seles | LH | 9 | 4 | 1990 | 1998 |
| Venus Williams | RH | 7 | 7 | 1997 | 2009 |
| Justine Henin | RH | 7 | 5 | 2001 | 2010 |
| Evonne Goolagong | RH | 5 | 11 | 1971 | 1977 |
| Martina Hingis | RH | 5 | 7 | 1997 | 2002 |
| Arantxa Sanchez-Vicario | RH | 4 | 8 | 1989 | 1998 |
| Hana Mandlikova | RH | 4 | 4 | 1980 | 1987 |
| Kim Clijsters | RH | 4 | 4 | 2001 | 2011 |
| Lindsay Davenport | RH | 3 | 4 | 1998 | 2005 |
| Maria Sharapova | RH | 3 | 2 | 2004 | 2011 |
| Jennifer Capriati | RH | 3 | - | 2001 | 2002 |
| Virginia Wade | RH | 3 | - | 1968 | 1977 |
| Mary Pierce | RH | 2 | 4 | 1994 | 2005 |
| Svetlana Kuznetsova | RH | 2 | 2 | 2004 | 2009 |
| Amelie Mauresmo | RH | 2 | 1 | 1999 | 2006 |
| Tracy Austin | RH | 2 | - | 1979 | 1981 |
| Jana Novotna | RH | 1 | 3 | 1991 | 1998 |
| Ana Ivanovic | RH | 1 | 2 | 2007 | 2008 |
| Ann Haydon Jones | LH | 1 | 2 | 1968 | 1969 |
| Conchita Martinez | RH | 1 | 2 | 1994 | 2000 |
| Gabriela Sabatini | RH | 1 | 2 | 1988 | 1991 |
| Kerry Reid | RH | 1 | 2 | 1970 | 1977 |
| Mima Jausovec | RH | 1 | 2 | 1977 | 1983 |
| Francesca Schiavone | RH | 1 | 1 | 2010 | 2011 |
| Li Na | RH | 1 | 1 | 2011 | 2011 |
| Nancy Richey | RH | 1 | 1 | 1968 | 1969 |
| Samantha Stosur | RH | 1 | 1 | 2010 | 2011 |
| Anastasia Myskina | RH | 1 | - | 2004 | - |
| Barbara Jordan | RH | 1 | - | 1979 | - |
| Chris O'Neil | RH | 1 | - | 1978 | - |
| Iva Majoli | RH | 1 | - | 1997 | - |
| Petra Kvitova | LH | 1 | - | 2011 | - |
| R.A. Cawley | RH | 1 | - | 1980 | - |
| Sue Barker | RH | 1 | - | 1976 | - |
| Virginia Ruzici | RH | 1 | - | 1978 | - |
| Helena Sukova | RH | - | 4 | 1984 | 1993 |
| Dinara Safina | RH | - | 3 | 2008 | 2009 |
| Mary Joe Fernandez | RH | - | 3 | 1990 | 1993 |
| Wendy Turnbull | RH | - | 3 | 1977 | 1980 |
| Andrea Jaeger | RH | - | 2 | 1982 | 1983 |
| Elena Dementieva | RH | - | 2 | 2004 | 2004 |
| Helen Gourlay | RH | - | 2 | 1971 | 1977 |
| Olga Morozova | RH | - | 2 | 1974 | 1974 |
| Renata Tomanova | RH | - | 2 | 1976 | 1976 |
| Rosemary Casals | RH | - | 2 | 1970 | 1971 |
| Sylvia Hanika | LH | - | 2 | 1980 | 1981 |
| Vera Zvonareva | RH | - | 2 | 2010 | 2010 |
| Anke Huber | RH | - | 1 | 1996 | - |
| Betsy Nagelsen | RH | - | 1 | 1978 | - |
| Betty Stove | RH | - | 1 | 1977 | - |
| Caroline Wozniacki | RH | - | 1 | 2009 | - |
| Dianne Fromholtz | LH | - | 1 | 1977 | - |
| Florenta Mihai | RH | - | 1 | 1977 | - |
| Helga Niessen | RH | - | 1 | 1970 | - |
| Jelena Jankovic | RH | - | 1 | 2008 | - |
| Judy Tegart | RH | - | 1 | 1968 | - |
| Kathy Jordan | RH | - | 1 | 1983 | - |
| Marion Bartoli | RH | - | 1 | 2007 | - |
| Natasha Zvereva | RH | - | 1 | 1988 | - |
| Nathalie Tauziat | RH | - | 1 | 1998 | - |
| Pam Shriver | RH | - | 1 | 1978 | - |
| Sharon Walsh | RH | - | 1 | 1979 | - |
| Zena Garrison | RH | - | 1 | 1990 | - |

For each female player who ever made it into a Grand Slam final in the open era (1968-2011) the table lists the full name, the hand used for playing tennis (LH = Left-handed player, RH = Right-handed player), the number of finals won and lost, and the first and the last year (only for players with more than one final played) a player was a Grand Slam finalist.
