# Supplementary material for: Left-Handedness in Professional and Amateur Tennis
Source: PLoS One. 2012 Nov 7;7(11):e49325. doi: 10.1371/journal.pone.0049325 (PMC3492260; doi:10.1371/journal.pone.0049325)
Supplement: Table S7 — Handedness in men’s and ladies’ amateur tennis across performance levels. (DOCX) [file pone.0049325.s007.docx]

**Table S7. Handedness in men’s and ladies’ amateur tennis across performance levels.**

|  | **Men** | | | **Ladies** | | |
| --- | --- | --- | --- | --- | --- | --- |
| **Performance Level** | **N** | **Left (%)** | **Right (%)** | **N** | **Left (%)** | **Right (%)** |
| 1 | 44 | 15.91 | 84.09 | 31 | 9.68 | 90.32 |
| 2 | 34 | 14.71 | 85.29 | 14 | 14.29 | 85.71 |
| 3 | 22 | 9.09 | 90.91 | 17 | 11.76 | 88.24 |
| 4 | 43 | 11.63 | 88.37 | 15 | 13.33 | 86.67 |
| 5 | 35 | 8.57 | 91.43 | 29 | 3.45 | 96.55 |
| 6 | 34 | 11.76 | 88.24 | 23 | 4.35 | 95.65 |
| 7 | 45 | 6.67 | 93.33 | 32 | 3.13 | 96.88 |
| 8 | 46 | 8.70 | 91.30 | 38 | 2.63 | 97.37 |
| 9 | 55 | 7.27 | 92.73 | 32 | 6.25 | 93.75 |
| 10 | 47 | 6.38 | 93.62 | 38 | 7.89 | 92.11 |
| 11 | 46 | 8.70 | 93.48 | 37 | 2.70 | 97.30 |
| 12 | 61 | 4.92 | 95.08 | 47 | 2.13 | 97.87 |
| 13 | 72 | 9.72 | 91.67 | 40 | 2.50 | 97.50 |
| 14 | 65 | 9.23 | 93.85 | 52 | 9.62 | 90.38 |
| 15 | 81 | 13.58 | 87.65 | 57 | 3.45 | 96.55 |
| 16 | 85 | 7.06 | 92.94 | 53 | 5.56 | 94.44 |
| 17 | 124 | 10.48 | 89.52 | 82 | 2.41 | 97.59 |
| 18 | 142 | 2.82 | 97.89 | 76 | 9.21 | 90.79 |
| 19 | 131 | 6.87 | 95.42 | 116 | 8.55 | 91.45 |
| 20 | 158 | 6.96 | 94.94 | 116 | 1.72 | 98.28 |
| 21 | 122 | 8.20 | 91.80 | 99 | 5.00 | 95.00 |
| 22 | 263 | 3.04 | 98.10 | 246 | 1.56 | 98.44 |
| 23 | 415 | 4.10 | 95.90 | 302 | 3.31 | 96.69 |

This table shows the handedness distribution in male and female amateur players across performance levels ranging from 1 (highest level) to 23 (lowest level). Note that the values listed here are based on the data we received from 184 tennis clubs (out of a total of 597 clubs).
